# Multifamily therapy in first episodes of psychosis: a pilot study

**DOI:** 10.1192/j.eurpsy.2022.2024

**Published:** 2022-09-01

**Authors:** I. García Del Castillo, S. López García, A. Pérez Balaguer

**Affiliations:** 1 Hospital El Escorial, Psychiatry, San Lorenzo de El Escorial, Spain; 2 Hospital Universitario Puerta de Hierro Majadahonda, Psychiatry, Majadahonda, Spain

**Keywords:** multifamily therapy, GROUP THERAPY, First episode of psychosis, early intervention

## Abstract

**Introduction:**

Multifamily interventions have shown to reduce the risk of relapse of psychotic symptoms in first episodes of psychosis (FEPs) but are not frequently implemented in specific treatment programs. We have develop a pilot study for the implementation of the interfamily therapy in FEPs within a Mental Health Centre in the Community of Madrid.

**Objectives:**

The aims were to examine: relapses (measured as re-hospitalization), duration of re-hospitalizations and voluntary versus involuntary re-hospitalizations during participation in MFG compared with the previous year.

**Methods:**

21 subjects participated in a MFG during 12 months, 11 participants with a diagnosis of psychosis and 10 family members. Interfamily therapy works as a new model of interactive psychoeducation among families where they share their own experiences and look for comprehension and solutions all together.

**Results:**

Our clinical experience in an interfamily therapy intervention over 12 months has led us to identify a high degree of participation and acceptance by users and their families, and we have observed a lower relapse rate, with fewer of psychiatric admissions and of shorter duration among patients during the year of participation in the MFG compared to the year before treatment.

**Conclusions:**

MFG has been well accepted by both patients and their families, with a high degree of participation.The results observed in our experience of MFG treatment are consistent with the findings of previous studies that support the reduction of the relapse rate, the number of hospitalizations and their duration when family interventions are incorporated into treatment in recent-onset psychosis, especially in a multi-family group format.

**Disclosure:**

No significant relationships.

